# Efficacy of Leuprolide Acetate 1-Month Depot for Central Precocious Puberty (CPP): Growth Outcomes During a Prospective, Longitudinal Study

**DOI:** 10.1186/1687-9856-2011-7

**Published:** 2011-07-12

**Authors:** Peter A Lee, E Kirk Neely, John Fuqua, Di Yang, Lois M Larsen, Cynthia Mattia-Goldberg, Kristof Chwalisz

**Affiliations:** 1 The Milton S. Hershey Medical Center, College of Medicine, Pennsylvania State University, Hershey, PA 17033, USA; 2 Section of Pediatric Endocrinology and Diabetology, James Whitcomb Riley Hospital for Children, School of Medicine, Indiana University, Indianapolis, IN 46202, USA; 3 Division of Pediatric Endocrinology and Diabetes, Room G313, Stanford University Medical Center, Stanford, CA 94305, USA; 4 Abbott Laboratories, 200 Abbott Park Road, Abbott Park, IL 60064, USA

## Abstract

**Introduction:**

Gonadotropin-releasing hormone analogs (GnRHa) are the treatment of choice for CPP. We investigated growth in GnRHa-naïve subjects, treated with leuprolide acetate 1-month depot for CPP.

**Methods:**

This prospective, open-label study had a long-term, observational, follow-up period. Forty-nine females and 6 males were enrolled. Leuprolide acetate depot was administered intramuscularly every 28 days. Height and growth rate during and after treatment until adulthood were measured.

**Results:**

Among 30 of 49 females having an adult height (AH) measurement, 29 had target heights available (mean = 163.8 cm) and 27 had pretreatment predicted adult heights (PAHs; mean = 157.4 cm). After treatment, the mean AH at mean age 21.8 years [range 13.7-26.7 years] was 162.5 cm, a mean height gain over baseline PAH of 4.0 cm. The mean height standard deviation score was -0.1 at AH.

**Conclusions:**

Treatment of CPP with leuprolide acetate 1-month depot had beneficial effects on growth rate and preservation of AH.

**Trial Registration:**

ClinicalTrials.gov: NCT00660010

## Introduction

Central precocious puberty (CPP) commonly refers to the development of pubertal sex characteristics as a consequence of the premature activation of the hypothalamic-pituitary-gonadal (HPG) axis before the age of 8 years in girls and 9 years in boys. The pathogenesis of CPP includes early activation of pulsatile release of gonadotropin-releasing hormone (GnRH), leading to an increase in secretion of gonadotropins and gonadal steroids. Central precocious puberty occurs much more frequently among females than males (greater than 20:1 ratio) [1].

The treatment goals for children with CPP include hormonal suppression, regression or cessation of development of pubertal characteristics and the prevention of short stature in adulthood. Patients with CPP are at risk of short stature in adulthood because of disproportionately advanced skeletal maturation in relation to growth acceleration, resulting in early epiphyseal fusion that limits growth potential [2].

Analogs of GnRH (GnRHa) have been the standard of care for the treatment of children with CPP for more than 20 years [2], with the usage of the depot form reported in 1989 [3]. However, despite the widespread use of GnRHa in the treatment of CPP, the factors that impact adult height outcomes after GnRHa therapy in children with CPP are poorly understood [4-13].

The few previous studies that have examined outcomes during and after treatment with leuprolide acetate have utilized variable treatment doses, and generally small numbers of patients [14-16]. The objectives of this longitudinal, prospective study were to evaluate the suppression of hormonal and clinical sexual characteristics and to examine the long-term impact of baseline and during-treatment factors on the growth pattern and adult height after treatment with leuprolide acetate 1-month depot in females with CPP. In this report, we describe the auxological outcomes of the study and analyze the effect of multiple baseline and during-treatment factors that potentially affect height outcomes in females. Though males were included in the study, firm conclusions regarding height outcomes cannot be drawn due to the small sample size. However, brief descriptive results have been included herein. A separate article reports the hormonal and reproductive outcomes of the study [17].

## Patients and Methods

### Patients

Patients were females with Tanner breast stage ≥2 before 8 years of age whose chronological age (CA) was less than 9 years. Males with Tanner genitalia stage ≥2 before 9 years of age whose CA was less than 10 years were also included. All had a baseline GnRH-stimulated LH level ≥10 IU/L. This level was chosen to ensure that all enrolled patients clearly had CPP, even though it was realized that some patients with CPP would be excluded. Additional inclusion criteria were bone age (BA) advancement ≥1 year over CA by the Fels Method [18], and treatment naïve to GnRHa. Institutional review board approval was obtained at each site, and written informed consent was provided by patients' parents or legal guardians prior to screening or any study related procedures.

### Study Design

This was a prospective, longitudinal, multicenter study that consisted of an open-label treatment period and a long-term post-treatment follow-up period. The study was conducted from 1991 to 2009 at nine U.S. centers. The primary outcomes of treatment in the study were the assessment of clinical sexual characteristics evaluated by Tanner staging and the decrease in serum gonadotropin and sex steroid concentrations to prepubertal levels. During the long-term follow-up into adulthood, the outcomes were adult height (based on stadiometer measurement or self-reported by questionnaire as adults) and information reported concerning reproductive system function.

Leuprolide acetate 1-month depot was started at a dose of 300 μg/kg (minimum starting dose of 7.5 mg) administered intramuscularly (IM) every 28 days. Incremental dose adjustments of 3.75 mg were made at each clinic visit, if necessary, based on the results of the previous GnRH stimulation test and hormonal parameters. GnRH stimulation tests for LH were performed by using a standard method (Factrel 100 μg IV bolus) in all patients. LH concentrations were measured by using DELFIA™ with a lower limit of quantitation of 0.15 IU/L.

Treatment period visits occurred at weeks 4, 8, 12, 24, 36, 48, and then every 6 months until study drug discontinuation. Study procedures included a physical examination with measurement of height and weight, assessment of Tanner stage, GnRH stimulation testing, and blood collection for gonadotropin and sex steroid levels. Bone radiographs were taken at baseline, week 24, week 48, and every 12 months thereafter. Radiographs were sent to a central reader (Wright State University School of Medicine, Lifespan Health Research Center, Kettering, OH) for determination of BA using the Fels Method [18]. Levels of gonadotropins and estradiol were determined by dissociation-enhanced lanthanide fluorescence immunoassay and radioimmunoassay, respectively (University of Pittsburgh Children's Hospital; Pittsburgh, PA). Target height was calculated by mid parental height plus or minus 6.5 cm for males and females, respectively; target height range was calculated by target height ± 8.5 cm. Predicted adult heights (PAH) were calculated by dividing a subject's height by the average percent of mature height associated with a given BA. The average percents of mature height were derived from tables for accelerated boys and girls prepared by Bayley and Pinneau [19]. Study drug was discontinued at the appropriate age for puberty at the discretion of the investigator.

After study drug withdrawal, patients entered into an optional follow-up period, with study visits occurring every 6 months until physical and laboratory measurements reached pubertal levels, and then annually until age 21 years. Follow-up study visits included a physical examination with measurement of height and weight, Tanner staging, basal levels of gonadotropins and sex steroids after evidence of pubertal response was observed with stimulated levels, menstrual and sexual history, and bone radiographs of the left hand and wrist. Adult height data were collected at adulthood (after age 18), during an office visit or by a patient-reported questionnaire (n = 19). For patients who were missing adulthood height data, adult height was established if the final height obtained during follow-up was associated with a growth velocity of <1 cm/year or a BA ≥14 years in females (n = 11) or ≥15 years in males.

### Statistics

Mean incremental growth rate was calculated as the ratio of the change in height to the change in CA at approximately six month intervals (1 month = 28 days to match dosing interval) during the treatment period and through the first year of the follow-up period, and at approximately one year intervals thereafter. Baseline growth rate was calculated as the growth rate during the year prior to the start of the treatment period.

The ratio of the change in BA during each one year interval to the change in CA during the one year interval was computed. Mean height and mean height standard deviation score (HtSDS) were calculated at each visit during the treatment and the follow-up periods and at adult height.

Pearson's correlation coefficients were calculated between baseline characteristics (CA, BA, BA-CA, time to treatment from onset of CPP, basal and peak LH, basal and peak FSH, growth rate, height SDS and target height), during-treatment variables (duration of treatment, average growth rate, the mean ratio of change in BA to change in CA, mean basal and peak LH, and mean basal and peak FSH), and end of treatment variables (CA, BA, BA-CA, growth rate, and height SDS) and gain over baseline PAH, height gain over end of treatment height, and at adult height. Dependent variables that were statistically significantly correlated at α = 0.05 level with each auxological outcome were entered into a stepwise multiple regression analysis to determine the best predictors (statistically significant at α = 0.05 level) of each auxological outcome.

## Results

### Female Patients

**Figure 1:**
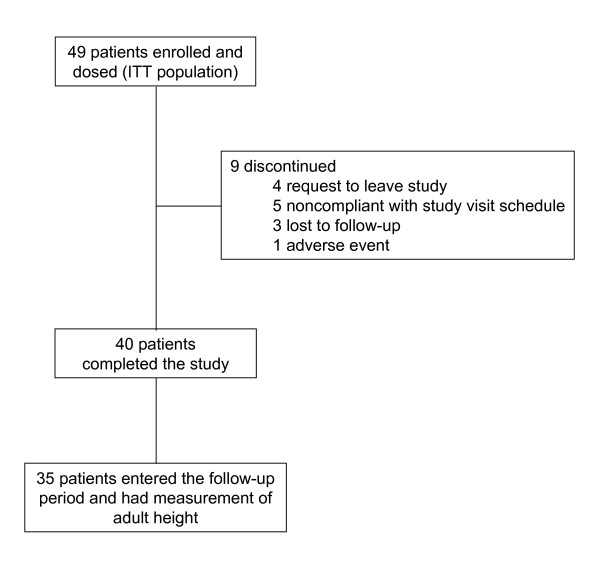
**Patient disposition**. Flow of female patients through the trial. Patients may have had more than one reason for treatment discontinuation.

This study consisted of a mean ± SD treatment period of 3.9 ± 2.0 years and a post-treatment follow-up period of 3.5 ± 2.2 years [17]. The study enrolled both females and males, however, too few males (n = 5) were enrolled to draw conclusions, and, therefore, were not included in the primary analysis presented in this report. Forty-nine females were enrolled in the treatment period and 35 of them participated in the long-term follow-up period (Figure 1). No premature discontinuations (n = 9) were related to lack of efficacy. All patients were naïve to treatment with GnRHa. Patient demographics and baseline characteristics for the female study population are summarized in Table 1. Overall, 61.2% of patients were Caucasian. At baseline, the mean age was 7.3 years and BA was advanced over CA by an average 3.0 years. Overall, the mean time since diagnosis of CPP was 0.3 years in the study population. All subjects had peak stimulated LH suppressed to <1.75 IU/L during treatment with leuprolide acetate 1-month depot, with 5 subjects requiring an increased dosage to obtain or retain suppression over the course of treatment. After discontinuation from study drug, the mean ± SD time to first menses was 1.5 ± 0.5 years (range of 0.5-2.5 years). The gonadotropin and sex steroid data are discussed in detail in a separate paper.

**Table 1:** Pretreatment subject demographics and baseline characteristics

	Female Patients
Characteristic	N = 49
Race, n (%)	
Caucasian	30 (61.2)
African American	11 (22.4)
Asian	0
Hispanic	8 (16.3)

Age, years	
Mean (SD)	7.3 (1.9)
Range	1.2-9.4

Weight, kg	
Mean (SD)	33.6 (9.72)
Range	13.0-52.2
Height, cm	
Mean (SD)	131.6 (14.97)
Range	84.6-154.7

Height Standardized Score	
Mean (SD)	1.5 (1.29)
Range	-1.4-3.4

Tanner Stage, n (%)	
Breast/Genitalia I	1 (2.0)^a^
II	9 (18.4)
III	25 (51.0)
IV	13 (26.5)
V	1 (2.0)

Growth Velocity, cm/yr	
Mean (SD)	10.7 (3.40)
Range	5.5-21.2

BA, years	
Mean (SD)	10.2 (2.13)
Range	2.5-12.1

BA - CA, years	
Mean (SD)	3.0 (0.80)
Range	1.3-4.5

History of Menstrual Bleeding, n (%)	
No	36 (75)
Yes	12 (25)

^a^A one year old patient was enrolled in the trial with breast Tanner stage I based on qualifying peak stimulated LH (84.7 IU/L) and E_2 _(90 pg/mL).

#### Growth During Treatment

**Figure 2:**
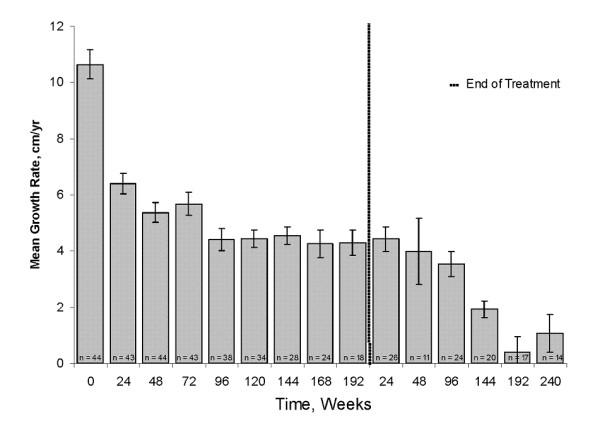
**Mean ± SE growth rates for females**. Incremental growth rates were calculated every 6 months. The baseline growth rate was defined as the growth rate for 1 year prior to the start of study drug. Numbers reflect available results.

The mean incremental growth rate was greatly reduced from 10.6 cm/year at baseline to approximately 5-6 cm/year during the first 72 weeks of therapy and then 4-4.5 cm/year from week 72 to week 192 (Figure 2). These mean growth rates during treatment were generally comparable to normal growth rates in prepubertal girls.

#### Post-treatment Growth

During the last 6 months of treatment, among patients who were enrolled into the follow-up period of the study, the mean incremental growth rate was 4.0 cm/year. Following withdrawal of study drug, mean incremental growth rate increased to 4.4 cm/year (Figure 2) at the 24 week follow-up visit, showing a small growth spurt, before gradually decreasing to 0.4 cm/year by follow-up week 192.

#### Bone Age and Predicted Height

**Figure 3:**
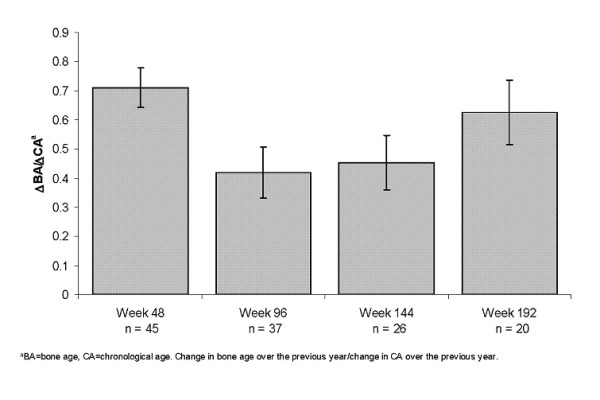
**Change in bone age to change in chronological age during each year of treatment for females**. The ratio of the change in BA from the previous visit to the change in CA from the previous visit was calculated at approximately one year intervals. Numbers reflect available results.

Prior to the initiation of treatment, mean BA advance over CA was 3 years. Treatment with leuprolide acetate 1-month depot rapidly controlled the advance in BA, with mean ΔBA/ΔCA of 0.7 after the first year of treatment, and not more than 0.6 in during the next 3 years of treatment (Figure 3).

#### Adult Height and Height Gain

**Figure 4:**
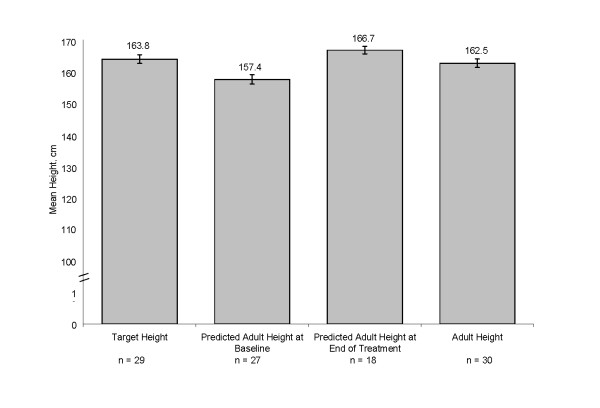
**Height outcomes for females during the study**. Adult height includes data from patients whose height was collected at adulthood after age 18. For patients who were missing adulthood height data, adult height was established when their growth velocity was <1 cm/year or bone age was ≥14 years during the follow-up period.

**Figure 5:**
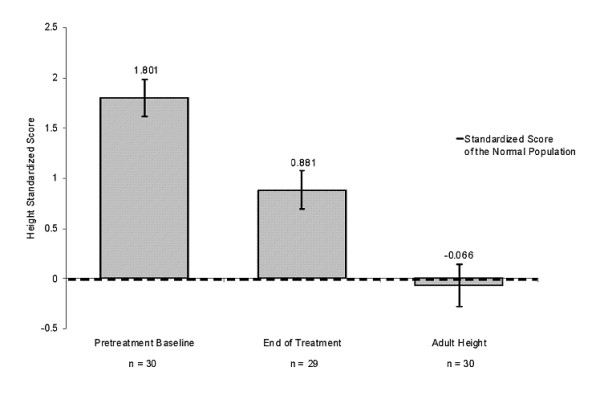
**Height standardized scores for females during the study**. The pretreatment baseline is the mean standardized height score of patients before initiation of leuprolide acetate therapy. The height at the end of treatment refers to the mean standardized height score for patients at the end of the treatment period. Adult height includes standardized scores for those patients whose height was collected at adulthood after age 18. For those without adult height available, adult height was established when their growth velocity was <1 cm/year or bone age was ≥14 years during the follow-up period.

Among the 30 females with adult height data, 29 females had a mean target height of 163.8 cm at baseline, and 27 subjects had a mean PAH of 157.4 cm at baseline. Of the 30 females with adult height data, 22 were measured with stadiometer, and 8 were self-reported. The mean adult height was 162.5 cm, which represents a mean height gain over baseline PAH of 4.0 cm. (Figure 4). In addition, mean height SDS was -0.1 at adult height, indicating that these patients essentially reached the mean height of the normal population by adulthood (Figure 5). Among the 29 females with menarche status at baseline and adult height, subjects who were premenarchal (n = 22) or post-menarchal (n = 7) had similar mean ± SD adult height outcomes (162.8 ± 6.77 cm vs 162.0 ± 1.6 cm, respectively).

#### Analysis of Factors Potentially Predictive of Height Outcomes

Univariate analyses were performed to identify factors that were strongly correlated with adult height, height gain over baseline PAH, and height gain after treatment (Tables 2, 3, and 4). After stepwise multiple linear regression of the factors that were significantly correlated in the univariate analysis, there were significant positive associations of average growth rate during treatment (p = 0.0002) and height SDS at baseline (p < 0.0001) with adult height (Table 2). For height gain over baseline PAH, BA minus CA at baseline (p = 0.0028) and time to treatment from onset of CPP (p = 0.0245) were found to be significantly positively and negatively associated, respectively (Table 3). Finally, for height gain after treatment, significant negative associations were found for BA at baseline (p < 0.0001) and duration of treatment (p = 0.0002, Table 4).

**Table 2:** Analysis of factors significantly associated with adult height in females

**Adult Height**^**a**^			
Univariate Analysis	Correlation Coefficient		P-value
**At the start of therapy**			
CA (yr)	-0.37		0.0348
BA (yr)	-0.38		0.0306
Peak FSH	0.35		0.0466
Growth rate	0.39		0.0336
Height SDS	0.82		<.0001
Target height	0.53		0.0019
**During therapy**			
Averaged growth rate	0.62		0.0001
**At the end of therapy**			
Height SDS	0.82		<.0001

**Multiple Linear Regression**	**Beta Coefficient**	**P-value**	**R**^**2**^

Average growth rate during treatment	3.05	0.0002	
Height SDS at baseline	5.11	<0.0001	0.82

^a^Adult height was established when the height was associated with a growth velocity of <1 cm/year or a bone age ≥14 years in females

**Table 3:** Analysis of factors significantly associated with gain over predicted adult height

**Gain Over Baseline Predicted Adult Height**^**a**^			
Univariate Analysis	Correlation Coefficient		P-value
**At the start of therapy**			
CA (yr)	-0.43		0.0221
BA minus CA	0.53		0.004
Time to treatment from onset of CPP (yr)	-0.39		0.0395
**During therapy**			
Duration of the treatment	0.52		0.0048
**At the end of therapy**			
CA	0.40		0.0369

**Multiple Linear Regression**	**Beta Coefficient**	**P-value**	**R**^**2**^

BA-CA at baseline	3.37	0.0028	
Time to Treatment from onset of CPP (yr)	-3.84	0.0245	0.41

^a^Adult height minus predicted mature height at baseline

**Table 4:** Analysis of factors significantly associated with height gain after treatment

**Height Gain After Treatment**^**b**^			
Univariate Analysis	Correlation Coefficient		P-value
**At the start of therapy**			
CA (yr)	-0.58		0.0005
BA (yr)	-0.73		<.0001
BA minus CA	-0.62		0.0001
Peak FSH	0.38		0.0344
Growth rate	0.44		0.0151
**During therapy**			
Duration of the treatment	0.39		0.0292
Averaged growth rate	0.58		0.0006
ΔBA/ΔCA	0.54		0.0015
Basal FSH	-0.36		0.0447
**At the end of therapy**			
BA	-0.63		0.0001

**Multiple Linear Regression**	**Beta Coefficient**	**P-value**	**R**^**2**^

BA at baseline	-3.08	<0.0001	
Duration of treatment	-1.87	0.0002	0.72

^a^Final adult height minus height at the end of treatment

### Male Patients

While auxological outcome analyses are not presented herein, the descriptive data on growth during treatment, post-treatment growth, and bone age and predicted height are presented below. A total of 6 males were enrolled in the treatment period, and 5 males participated in the long-term follow-up period. At baseline, the mean age of the 6 males was 7.5 years and on average, BA was advanced over CA by 3.2 years. Treatment with leuprolide acetate 1-month depot rapidly controlled the advancement in BA, with mean ΔBA/ΔCA of 1.2 after the first year of treatment. Mean growth rate during treatment was reduced from 9.6 cm/year at baseline to 4.5-9 cm/year during the first 72 weeks and 4.5-6 cm/year from week 72 through week 192, respectively. The mean incremental growth rate was 2.0 cm/year at the end of treatment; however, following withdrawal of study drug, the rate increased to 4.5 cm/year at the 24-week follow-up visit. The mean final adult height (n = 3) during follow-up was 4.7 cm less than the PAH at baseline. None of the male patients had final adult heights recorded at adulthood (after age 18).

## Discussion

The results of this prospective, longitudinal study show that leuprolide acetate 1-month depot effectively suppressed the advanced growth rate and rate of bone maturation in females with CPP who were naïve to treatment with GnRHa, similar to the effects observed in previous studies [20-24]. After discontinuation of study drug, a small increase in growth rate was observed during the first year post-treatment, suggesting the occurrence of a limited pubertal growth spurt. Furthermore, on average, patients in this study population reached adult height of the standard normal population after treatment with leuprolide acetate 1-month depot.

Using the Bayley-Pinneau method at baseline to predict the adult height of our study population, adult heights were on average 4.0 cm greater than PAH at onset of treatment with leuprolide acetate among female patients. This is consistent with previous studies using depot GnRHa for CPP, which have demonstrated that females between the ages of 6-8 years have a moderate height increase that ranges from 4.5 cm to 7.2 cm [6,25]. While studies have failed to show benefit if treatment is begun after age 8 years [26], this does not take into account subsequent diminution of growth potential without treatment. Some studies suggest that the Bayley-Pinneau method may over-predict adult height among those with advanced skeletal age by several centimeters at baseline [2,7,27]. The 30 patients who had adult heights in this study had a mean HtSDS of -0.1, which indicates that their mean height was within the range of target heights equivalent to that of the normal population. Twenty-four of the 29 patients with target heights reached their target height range.

Similar to previous studies (11, 15, 19, 21, 22, 26-28), we used multiple linear regression to identify factors that may affect height gain from baseline and from the end of treatment and final adult height in females with CPP. Factors having significant correlation by multiple linear regression analyses (Tables 2, 3, and 4) are discussed below.

### Factors Influencing Adult Height

The two factors that explained 82% of the variability in adult height were HtSDS score at baseline and average growth rate during treatment. Both were positively correlated and similarly identified as positive factors in a previous study in determining patients who may benefit the most with regard to height gain from leuprolide acetate 1-month depot therapy [20]. The positive correlation between growth rate during treatment and adult height highlights the importance of monitoring the level of growth rate suppression during treatment Adequate growth rates during treatment were associated with positive final growth outcomes in our study population. The growth rates observed in this study (5-6 cm/year during the first 72 weeks of therapy and 4-4.5 cm/year from week 72 to week 192) were similar to growth rates observed in normal prepubertal children of these ages. Unlike prior reports and common expectations, CA at onset of therapy did not predict height outcome. This is likely related to the diversity of age at onset of puberty.

### Factors Influencing Height Gain Over PAH at Baseline

In the multiple linear regression for height gain over PAH at baseline, the advancement of BA over CA at baseline was a strong positive predictor, and time to treatment from onset of CPP was a strong negative predictor. This indicates that the greater the advancement of BA over CA at baseline and the shorter the interval between onset of CPP and the initiation of treatment, the greater the height gain (defined as adult height minus predicted mature height at baseline). This observation is similar to results reported by Mul et al. [12]. Overall, the advancement of BA over CA at baseline and the time to treatment from onset of CPP accounted for 41% of the variability in height gain. The finding that advancement in BA at the onset of therapy is a positive predictor of height gain over PAH may seem inconsistent with the previously described inverse relationship of BA at onset of therapy and adult height [20]. Because subjects with a more advanced BA have a lower PAH at the onset of therapy, halting advancement of BA during GnRHa therapy appears to result in a greater gain in height than the initial predicted height. This would explain the positive predictor of height gain, but not actual adult height. Hence, there appears to be a greater "gain back" growth potential among those in this study having more advanced BAs at onset of therapy.

### Factors Influencing Height Gain After Stopping Therapy

The duration of therapy had a negative association with the height gain after stopping therapy. Previous studies have shown no association between duration of therapy and height gain after therapy [20,25]. The negative correlation between BA at baseline and height gain after stopping therapy is expected, and was observed by Brito et al [20]. The two factors of treatment duration and BA at baseline explain 72% of the variance in height gain after treatment. The univariate analysis showing a correlation between ΔBA/ΔCA and growth after therapy does not persist with the multiple linear regression analyses; hence, this is not a major factor. The difference in the results between the univariate and multiple linear regression analyses might be explained by the considerable variation in BAs. At the onset of therapy, BA would be expected to continue to advance until typical pubertal BA is reached. The ratio, therefore, may reflect considerable variation.

In summary, treatment with leuprolide acetate 1-month depot increased adult height over the PAH. In addition, the HtSDS was equivalent to that of the normal population, indicating that these patients had on average nearly achieved the height of their peers without CPP. Overall, treatment with leuprolide acetate 1-month depot preserved adult height potential and resulted in clinically meaningful height gains over PAH. The results of this study confirm that in females, early initiation of treatment from the onset of CPP results in the greatest increase in height. In addition, female patients with the greatest advance in BA experience the most height gain over PAH with GnRHa treatment, and maintaining growth rates within that of normal prepubertal children during GnRHa treatment may result in the highest adult height outcome.

## Competing interests

PAL has been a consultant for Abbott and Novo Nordisk with grants from Abbott, Eli Lilly, Novo Nordisk, Pfizer, and Ipsen. EKN has received grant/research support from and has been a consultant and scientific advisory board member for Abbott. JF has been a scientific advisory board member for Abbott. DY, LL, CMG, and KC are all employees of Abbott and LL, CMG, and KC all own Abbott stock.

## Authors' contributions

PL was involved in the original protocol, the laboratory assessments, the planning and execution of the phase 4 (follow-up) study as well as the preparation of the manuscript. EKN and JF participated in data collection and interpretation and manuscript preparation. LL and DY performed the statistical analysis and participated in drafting the manuscript. CMG and KC participated in interpreting the data and manuscript preparation. All authors read and approved the final manuscript.
